# Apoptotic Changes, Oxidative Stress and Immunomodulatory Effects in the Liver of Japanese Seabass (*Lateolabrax japonicus*) Induced by Ammonia-Nitrogen Stress during Keep-Live Transport

**DOI:** 10.3390/biology12060769

**Published:** 2023-05-25

**Authors:** Meijie Guo, Qi Yan, Yixuan Dong, Zhaoyang Ding, Jun Mei, Jing Xie

**Affiliations:** 1 College of Food Science and Technology, Shanghai Ocean University, Shanghai 201306, China; m210311056@st.shou.edu.cn (M.G.); m210311057@st.shou.edu.cn (Y.D.); zyding@shou.edu.cn (Z.D.); 2 National Experimental Teaching Demonstration Center for Food Science and Engineering, Shanghai Ocean University, Shanghai 201306, China; m210311047@st.shou.edu.cn; 3 Shanghai Engineering Research Center of Aquatic Product Processing and Preservation, Shanghai 201306, China; 4 Shanghai Professional Technology Service Platform on Cold Chain Equipment Performance and Energy Saving Evaluation, Shanghai 201306, China

**Keywords:** Japanese seabass, ammonia-nitrogen stress, keep-live transport, oxidative stress, immunological response

## Abstract

**Simple Summary:**

Excreta produced by fish during preservation transport can increase ammonia-nitrogen (NH_3_-N) levels in transport water and reduce survival rates. However, studies on the effects of NH_3_-N stress on Japanese seabass during transport are limited. The objective of this study was to investigate the effects of NH_3_-N stress on tissue damage, oxidative stress, apoptosis and immunity in Japanese seabass during transport, and to determine tolerance and response to NH_3_-N toxicity. The results of this study provide insight into the impact of environmental factors on Japanese seabass during maintenance of live transport, which can be considered during transport in terms of improving the quality of transport water, among other things, to develop optimal procedures for live fish transport and improve survival rates during transport.

**Abstract:**

This study investigated the effects of NH_3_-N on antioxidant responses, histoarchitecture, and immunity of Japanese seabass (*Lateolabrax japonicus*) during keep-live transport. The findings suggest that NH_3_-N stress transport alters the transcription of *P53*, *Caspase 9*, *Bcl2*, *Caspase 3* and *Bax* genes, demonstrating that NH_3_-N stress can trigger the apoptotic pathway of *P53*-*Bax*-*Bcl2* and Caspase and induce apoptosis. NH_3_-N stress transport also evoked transcriptional upregulation of inflammatory cytokines (*tumor necrosis factor α (TNF-α)*, *Toll-like receptor 3 (TLR-3)*, *nuclear factor kappa β (NF-κB)*, *interleukin 6 (IL-6) and interleukin 1β (IL-1β)*) and increased complement C3, C4, lysozyme (LZM) and immunoglobulin (IgM) levels, activating the innate immunological system during keep-live transport. In addition, NH_3_-N stress transport altered changes in the levels of superoxide dismutase (SOD), catalase (CAT), glutathione-related enzymes, and heat shock proteins 70 and 90 in the liver, indicating that the antioxidant system and Hsp protected the cells from NH_3_-N-induced oxidative stress. When excess ROS were not removed, they caused the body to respond with immunological and inflammatory responses, as well as apoptosis and tissue damage. This helps towards understanding the effect of NH_3_-N levels on sea bass during keep-live transport.

## 1. Introduction

In China, Japanese seabass (*Lateolabrax japonicus*) is an economically important cultured fish. Nevertheless, high-density seabass keep-live transport causes ammonia-nitrogen (NH_3_-N) concentration to rise to dangerous levels in a short period, endangering the life of the sea bass. Higher NH_3_-N levels can disturb the metabolism of organisms, cause tissue damage, lead to a decrease in immunity and the inflammatory reaction, and even kill fish [1]. At the same time, when fish have been in water with a high NH_3_-N content, this will lead to the continuous accumulation of reactive oxygen species (ROS), causing oxidative stress and metabolic disorders in the biological system of fish. To combat this issue, cells form antioxidant defense systems such as the glutathione system [2,3]. The dynamic balance of GSH/GSSG is critical to the organism’s defense and proper physiological condition [4]. Systematic analysis of the changes and functional regulation of glutathione in antioxidant defense can provide further insight into its role in NH_3_-N stress transport. In addition, heat shock protein (Hsp) is crucial in defending the organism from stress-related damage. Hsp protects cells from stressor-induced protein dysfunction, such as ROS and hypoxia [5]. The organism’s ability to tolerate NH_3_-N is tightly correlated with the expression pattern and levels of Hsp [6]. Hsp levels are raised as a result of NH_3_-N stress, according to earlier research [7].

Currently, there are many studies on NH_3_-N stress, transport stress and combined stress of various stressors. According to Tao et al. [8], hybrid yellow catfish (*Tachysurus fulvidraco* ♀ × *Pseudobagrus vachellii* ♂) activates tlr5 in the TLR signaling pathway during transport, which affects immunological and inflammatory responses. It was also found that heat stress transport reduced the content of antioxidant enzymes including CAT, T-AOC and meat quality in rainbow trout (*Oncorhynchus mykiss*) [9]. Dual stress tends to affect organisms more adversely than a single stressor. Molayemraftar et al. [10] discovered that the combined impact of NH_3_-N and nitrite is harmful to Common carp (*Cyprinus carpio*) antioxidant defenses and has negative effects on the blood. Esam et al. [11] also found that the combined stress of NH_3_-N and heat affect the Nile tilapia (*Oreochromis niloticus*) more in terms of immunosuppression, oxidative stress and inflammation.

However, there is little research on how NH_3_-N stress affects seabass during keep-live transport. The goal of this study was to look into the impact of NH_3_-N stress on tissue damage, oxidative stress, apoptosis and immunity of Japanese seabass during keep-live transport, as well as to identify the tolerance and response to NH_3_-N toxicity in terms of gene expression associated with the glutathione system and the *NF-κB* signaling pathway.

## 2. Materials and Methods

### 2.1. Experimental Fish

Japanese seabass (mean weight 400 ± 3.5 g, mean length 35 ± 1.0 cm) were obtained from the seafood market in Shanghai. The seabass were temporarily incubated in a 300 L food-grade tank in the laboratory for 36 h before transport experiments. Temporary incubation conditions were as follows: the water temperature was 20 °C, salinity was 16‰, pH was 7.5 and dissolved oxygen (DO) concentration was above 7.0 mg/L. To maintain high water quality, the water was replaced every day. After the transient period, the water was cooled from 20 to 12 °C at a rate of 3 °C/h.

### 2.2. Experiment to Determine LC_50_ and Experimental Procedure:

A NH_4_Cl master mix was used during the experiment to adjust the NH_3_-N concentration to reach the required value for each experimental group. The range of NH_3_-N concentration was obtained from the lowest concentration where no fish died in 48 h (2 mg/L) to the highest concentration where all fish died (40 mg/L) by the pre-experiment and divided logarithmically. Six concentration gradients were established—2 mg/L, 3.6411 mg/L, 6.6289 mg/L, 12.0684 mg/L, 21.9712 mg/L, and 40 mg/L. Nine fish were used in each experimental group and this was repeated three times. The number of dead Japanese seabass was recorded on the 12th, 24th, 36th and 48th h. Dead fish were removed promptly (considered dead if they were stationary and did not respond to the surrounding stimuli) and water quality management was the same as during the transient period. The LC_50_ = 21.32 mg/L at 48 h was calculated based on the survival rate.

LC_50_ = 21.32 mg/L was obtained for sea bass by pre-experiment. Four groups were set up in the formal experiment: a control group (CK), a low NH_3_-N group (LA, 6.4 mg/L), a medium NH_3_-N group (MA, 12.8 mg/L), and a high NH_3_-N group (HA, 19.2 mg/L), which were equivalent to 30%, 60% and 90% of the 48 h LC50, respectively (Table 1). NH_3_-N concentration was measured using an NH_3_-N detector (DWS-296, Shanghai Insa Scientific Instruments Co., Ltd., Shanghai, China) to achieve the specified NH_3_-N levels. The fish were reared in sealed tanks with a fish-to-water ratio of 1:2. The dissolved oxygen (DO) concentration was guaranteed to exceed 7.0 mg/L, the water temperature was 20 °C, salinity was 16‰ and pH was 7.5 during transport. Live fish were simulated transport in a vibration conveyor in terms of 1 h on a class B road, followed by 5 h on a class A road. The cycles were repeated 8 times [12]. The numbers of dead fish were recorded at 0, 12th, 24th, 36th and 48th h, respectively. After 48 h transport, the surviving fish recovered for 24 h in seawater under transient conditions.

### 2.3. Sample Collection and Processing

Samples were taken once at the 0, 12th, 24th, 36th, and 48th h of NH_3_-N exposure and at the 12th and 24th h of recovery, respectively. Eighteen fish were sampled per experimental group at a time, for a total of three replicate groups, using nine fish per replicate. The livers of sea bass were immediately removed, washed with saline and divided into two parts. One part of the liver tissue samples were immersed for 24 h and fixed for histological and TUNEL assays, the remaining samples were stored at −80 °C qPCR index determination and enzyme index measurement.

### 2.4. Biochemical, Immunological and Oxidation Parameters Analysis

Liver tissue samples were diluted 10 times with 0.1 M PBS and homogenized, then centrifuged at 5000× *g* for 10 min at 4 °C. The supernatant was extracted and used to determine the following indicators using commercial kits (Jiancheng Bioengineering Institute, Nanjing, China) measuring the following enzymes: SOD (A001-3-2), CAT (A001-3-2), alanine aminotransferase (ALT, C009-2-1), GST, aspartate aminotransferase (AST, C010-2-1), GSH-PX (A005-1-2), GR (A001-3-2), GSH/GSSG (A061-2-1), LZM (A050-1-1), total protein (TP, A045-2-2), immunoglobulin (IgM, H109-1-1), complement C3 (E032-1-1), complement C4 (E033-1-1), Hsp70 (H264-2-2), and Hsp90 (H264-3).

Malondialdehyde (MDA) in the liver was measured using the TBA method according to the kit (A003-1-2, Jiancheng Bioengineering Institute, Nanjing, China) instructions and in nmol/mg protein. MDA is a peroxidized lipid degradation product that condenses with thiobarbituric acid (TBA) to form a red product with a maximum absorption peak of 532 nm.

The level of ROS in the liver was detected with fluorescent probe 2′,7′-dichlorofluorescein diacetate (DCFH-DA) (E004-1-1, Jiancheng Bioengineering Institute, Nanjing, China). DCFH-DA can be oxidized by esterase into strong green fluorescent substance DCF (2′,7′-dichlorofluorescein) in the presence of reactive oxygen species. The liver tissue was homogenized and made into a single cell suspension. 2′,7′-dichlorofluorescein yellow diacetate was added to the suspension and then incubated for 1 h at 37 °C, centrifuged for 10 min, and single cells were collected and washed in PBS solution. The excitation wavelength of 500 nm and the fluorescence wavelength of 525 nm were used to determine the amount of ROS. These values were expressed as the following equation:

$$ROS\;content\;\left(\%\right)=\frac{fluorescence\;intensity\;of\;treated\;cells}{fluorescence\;intensity\;of\;control}\times 100$$

### 2.5. Apoptosis Detection

Samples were processed according to the method of He et al. [13]. Paraffin sections were made from liver tissue preserved in 4% formaldehyde solution. After dewaxing, hydration and cell permeation, the sections were assayed using a kit (Roche, Basel, Switzerland). TUNEL-positive cells were observed using a fluorescence microscope (Nikon, Tokyo, Japan). Positive apoptotic nuclei are green.

### 2.6. Histological Examination

The liver was dried, translucent, and embedded after being fixed for 24 h, and it was then sliced into 6 m slices using a slicer (Leica, Wetzlar Germany). The liver was examined under a light microscope (Leica, Wetzlar, Germany) after being stained with hematoxylin and eosin (HE). Then, the severity score is assigned to the degree and range of change: (−) no histopathology; (−) no histopathology; (+) <20% histopathology in the visual field; (++) 20–60% histopathology in the visual field; (+++) >60% histopathology in visual field [14].

### 2.7. Real-Time PCR

Total RNA was extracted from the liver sample using an RNA extraction solution (Xavier Biotechnology Co., Ltd., Wuhan, China). Ultramicro spectrophotometer (NanoDrop2000, Thermo Fisher Scientific, Waltham, MA, USA) was used to measure the quantity and quality of RNA. Using 10 μL of total RNA (200 ng/μL), the single-stranded cDNA was created by the directions of Servicebio^®^RT cDNA Synthesis Kit (G3330) (Xavier Biotechnology Co., Wuhan, China). The mRNA expression level of inflammatory factors and apoptotic genes was determined using RT-PCR. The total volume of the amplification reaction, which contained 2.0 μL of retrotranscription product (cDNA), 1.5 μL of 2.5 μM gene primer, 4.0 μL of water nuclease-free, and 7.5 μL of 2× qPCR Mix, was 15 μL. The reaction parameters were shown in Table 2. Primer sequences were designed according to the previously reported sequences (Table 3), and the 2^−ΔΔct^ technique was used to assess the degree of gene expression [15]. All samples were set in triplicate.

## 3. Results

### 3.1. Oxidative Stress Response

**Figure 1:**
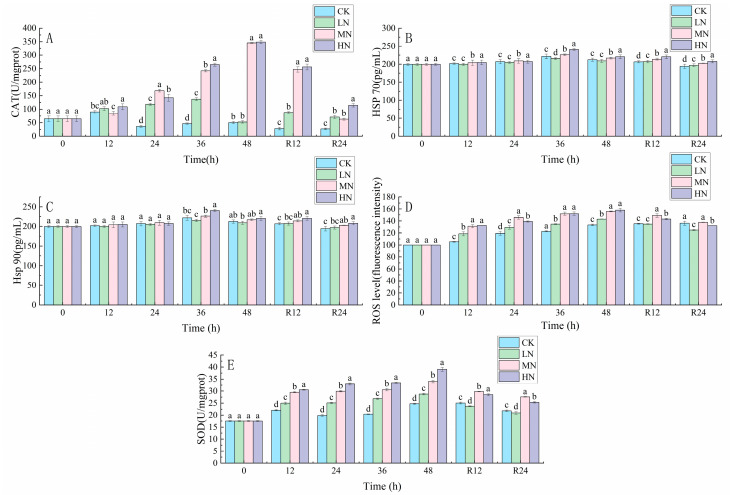
Changes in catalase (CAT) (**A**), heat stress protein 70 (Hsp 70) (**B**), heat stress protein 90 (Hsp90) (**C**), reactive oxygen species (ROS) (**D**) and superoxide dismutase (SOD) (**E**) concentrations in the liver of Japanese seabass exposed to NH_3_-N of 6.4 mg/L (LA), 12.8 mg/L (MA), 19.2 mg/L (HA), and 0 mg/L (CK) for 48 h and recovered for 24 h, respectively. Values are expressed as the mean ± S.D. Different lowercase letters indicate significant differences (*p* < 0.05) among groups. The CK NH_3_-N group served as the control. (*p* < 0.05, *N* = 3).

The CAT, SOD, and ROS contents of each sample increased continuously with the transportation time, and the MN and HN groups had higher CAT, SOD, and ROS contents after 48 h of transportation (Figure 1). The CAT, SOD, and ROS contents of all groups decreased to the initial levels. The Hsp 70 and Hsp 90 contents of each group increased with the transport time and reached the peak after 36 h of transport, and the Hsp 70 and Hsp 90 contents of the MN and HN groups were significantly higher than those of the CK group (*p* < 0.05).

### 3.2. Glutathione-Related Reactions

**Figure 2:**
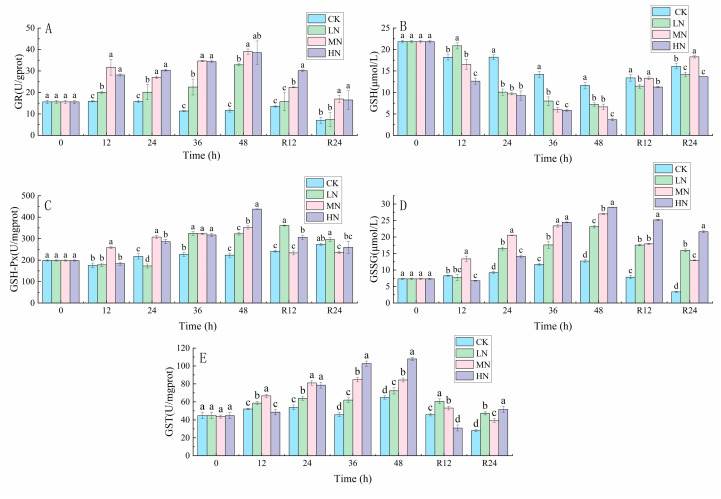
Changes in glutathione reductase (GR) (**A**), glutathione (GSH) (**B**), glutathione peroxidase (GSH-Px) (**C**), oxidized glutathione (GSSG) (**D**) and glutathione S-transferase (GST) (**E**) contents in the liver of Japanese seabass exposed to NH_3_-N of 6.4 mg/L (LA), 12.8 mg/L (MA), 19.2 mg/L (HA), and 0 mg/L (CK) for 48 h and recovered for 24 h, respectively. Values are expressed as the mean ± S.D. Different lowercase letters indicate significant differences (*p* < 0.05) among groups. The CK NH_3_-N group served as the control. (*p* < 0.05, *N* = 3).

During the recovery process, GR and GSSG contents in ammonia stress-treated samples showed a decreasing trend, while GSH contents showed an increasing trend. The contents of GSH-Px and GST in the liver of the NH_3_-N stress group increased with the transport time and reached the peak at 48th h. The contents of GSH-Px and GST decreased with the recovery time (Figure 2).

### 3.3. Tissue Damage and Liver Pathology

**Figure 3:**
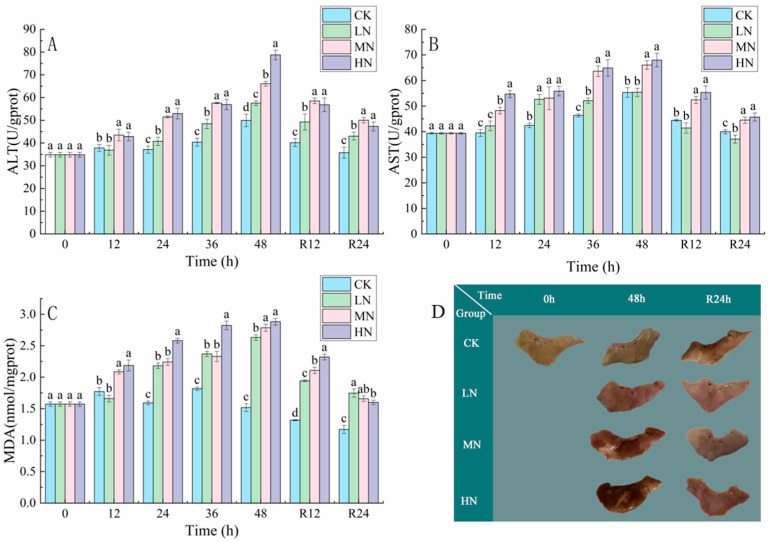
Changes in alanine aminotransferase (ALT) (**A**), aspartate aminotransferase (AST) (**B**), malondialdehyde (MDA) (**C**) and morphological of the liver (**D**) contents in the liver of Japanese seabass exposed to NH_3_-N of 6.4 mg/L (LA), 12.8 mg/L (MA), 19.2 mg/L (HA), and 0 mg/L (CK) for 48 h and recovered for 24 h, respectively. Values are expressed as the mean ± S.D. Different lowercase letters indicate significant differences (*p* < 0.05) among groups. The CK NH_3_-N group served as the control. (*p* < 0.05. *N* = 3).

The results in Figure 3 show that the ALT, AST, and MDA contents of all groups increased continuously with the transportation time, and the ALT, AST, and MDA contents of the HN group were the highest after 48 h of transportation (*p* < 0.05), and the ALT, AST, and MDA contents of all groups showed a decreasing trend in the recovery period.

The liver color of the LN, MN, and LN groups was significantly darker than that of the CK after 48 h of NH_3_-N stress transport, especially the LN group. After 24 h of recovery, the livers of the LN, MN, and LN groups were significantly lighter compared to the 48 h of transport, but still darker compared to the CK (Figure 3).

**Figure 4:**
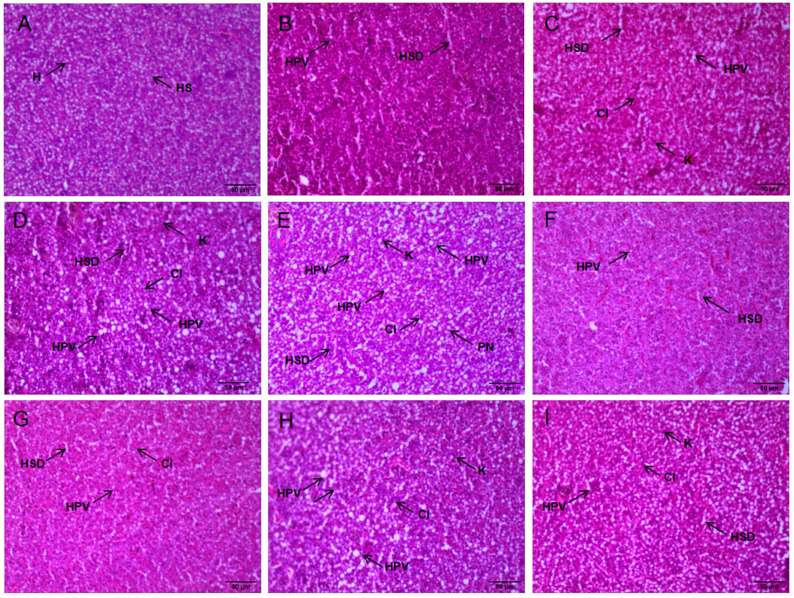
Paraffin sections of liver tissue were stained with HE to assess the pathological changes in different NH_3_-N concentrations on Japanese seabass. Micrographs (×400) and scale bars (50 μm). (**A**) represents tissue sections of the normal liver at 0 h; (**B**–**E**) represents liver tissue sections of 6.4 mg/L (LA), 12.8 mg/L (MA), 19.2 mg/L (HA), and 0 mg/L (CK) at 48th h after NH_3_-N stress transport; (**F**–**I**) represents liver tissue sections of 6.4 mg/L (LA), 12.8 mg/L (MA), 19.2 mg/L (HA), and 0 mg/L (CK) at 24th h after recovery from NH_3_-N stress transport. The CK NH_3_-N group served as the control. (H, hepatocyte; VD, vacuolar degeneration; HS, hepatic sinusoid; HSD, hepatic sinusoid dilatation; K. karyolysis; HPV, hepatocellular vacuolation; PN, cellular peripheral nucleus; CI, cellular outline indistinguishable).

In addition, histopathological damage of the seabass liver occurred to different degrees after 48 h of NH_3_-N stress transport (Table 4). The CK liver showed normal hepatocytes with the neat arrangement, normal morphology, clear and intact boundaries, and nuclei located in the center of the cells (Figure 4). Hepatic tissues in the LN and MN groups showed hepatocyte vacuolization and hepatic sinusoidal dilatation compared with the CK (Table 4). Liver tissues in the HN group were more severely damaged, showing more hepatocyte vacuolization and hepatic sinusoidal dilatation, and the nuclei shifted to the periphery and the cell boundaries blurred.

### 3.4. Immune Response and Inflammatory Factors

**Figure 5:**
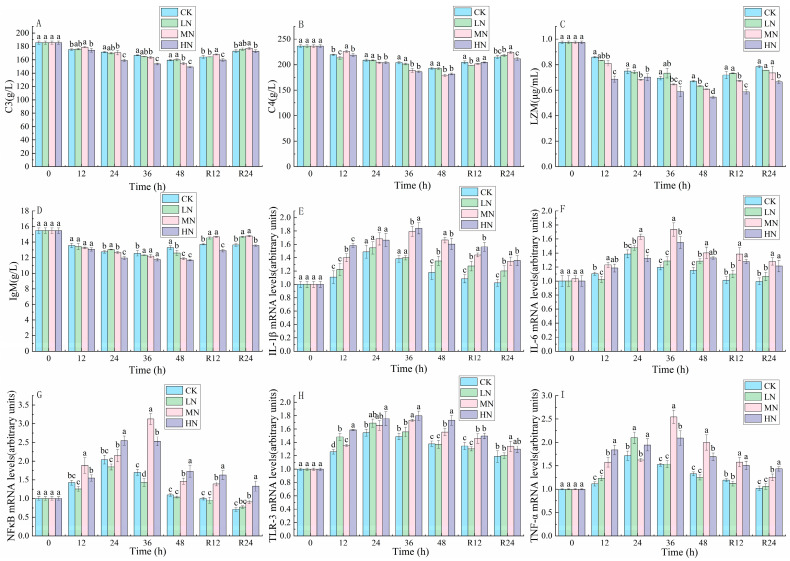
Changes in complement C3 (**A**), complement C4 (**B**), lysozyme (LZM) (**C**), immunoglobulin (IgM) (**D**), interleukin 1β (IL-1β) (**E**), interleukin 6 (IL-6) (**F**), nuclear factor kappa β (NF-κB) (**G**), Toll-like receptor 3 (TLR-3) (**H**) and tumor necrosis factor α (TNF-α) (**I**) contents in the liver of Japanese seabass exposed to NH_3_-N of 6.4 mg/L (LA), 12.8 mg/L (MA), 19.2 mg/L (HA), and 0 mg/L (CK) for 48 h and recovered for 24 h, respectively. Values are expressed as the mean ± S.D. Different lowercase letters indicate significant differences (*p* < 0.05) among groups. The CK NH_3_-N group served as the control (*p* < 0.05, *N* = 3).

The results in Figure 5 show that the contents of C3, C4, IgM, and LZM decreased continuously with the transport time. The contents of C3, C4, IgM, and LZM increased during the recovery process. After 24 h of recovery, these parameters were still lower in the HN group than in the CK.

The expressions of *TLR-3*, NFκB, *TNF-α*, *IL-6* and 1L-1β were first increased and then decreased in all groups. With the increase in NH_3_-N transport stress time, the expression of *TLR-3*, NFκB, *TNF-α*, *IL-6* and 1L-1β in the CK and LN groups peaked at 24th h, while the gene expression of each pro-inflammatory factor in the MN and HN groups peaked at 36th h (Figure 5).

### 3.5. Apoptosis

**Figure 6:**
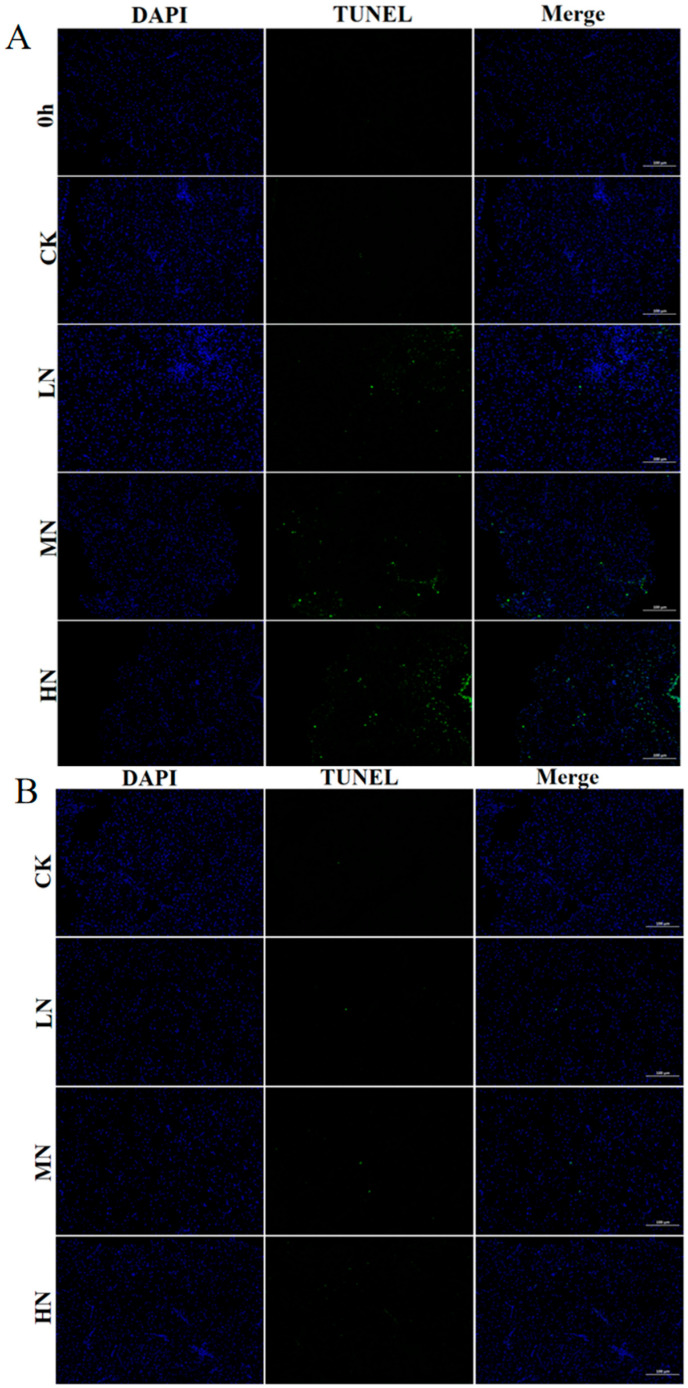
The TUNEL staining results of liver tissues of Japanese marine fish exposed to 6.4 mg/L (LA), 12.8 mg/L (MA), 19.2 mg/L (HA) and 0 mg/L (CK) NH_3_-N concentrations in transport water at 0th h and 48th h (**A**) and after 24 h of recovery (**B**). The CK NH_3_-N group served as the control.

As shown in Figure 6, positive TUNEL signals were detected in all groups after 48 h of NH_3_-N stress transport. The number of apoptotic cells was HN < MN < LN < CK. After 24 h of recovery, the number of positive cells declined in each group.

**Figure 7:**
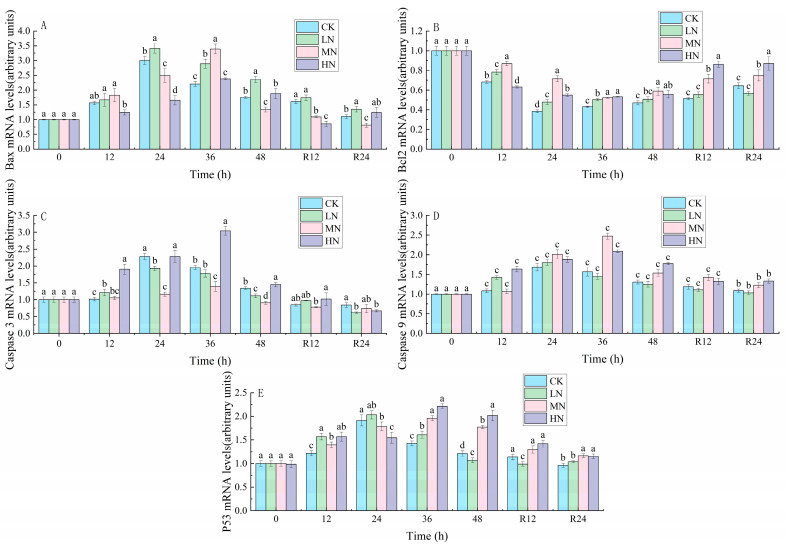
Changes in *Bax* (**A**), *Bcl2* (**B**), *Caspase 3* (**C**), *Caspase 9* (**D**) and *P53* (**E**) contents in the liver of Japanese seabass exposed to NH_3_-N of 6.4 mg/L (LA), 12.8 mg/L (MA), 19.2 mg/L (HA), and 0 mg/L (CK) for 48 h and recovered for 24 h, respectively. Values are expressed as the mean ± S.D. Different lowercase letters indicate significant differences (*p* < 0.05) among groups. The CK NH_3_-N group served as the control. (*p* < 0.05, *N* = 3).

The peak expression levels of *P53*, *Caspase 3*, *Caspase 9* and *Bax* genes in the CK and LN groups appeared at 24th h, while the peak expression levels of *P53*, *Caspase 3*, *Caspase 9* and *Bax* genes in the CK and LN groups appeared at 36th h (Figure 7).

## 4. Discussion

### 4.1. The impact of NH_3_-N Stress Transport on the Antioxidant Defense System of the Liver of Japanese Seabass

ROS are increased in concentration by ammonia, which causes oxidative stress in living things [1]. Organisms reduce ROS by increasing the levels of antioxidant enzymes (e.g., CAT, GST and SOD) to reduce ROS-induced oxidative damage. The initial line of defense in preventing the harm that oxidative stress causes is thought to be SOD-CAT and SOD-GPX [22]. Superoxide is converted to H_2_O_2_ by SOD, which is then reduced to water by CAT or GPX [23]. Because it maintains metabolic homeostasis and removes toxins, the liver is the ideal organ for assessing oxidative stress [24]. ROS produced by Japanese seabass under NH_3_-N stress transport could activate its antioxidant defense system, which prevented the damage caused by NH_3_-N and transport stress to the organism. A similar finding was made by Cheng et al. [1], where excessive ROS production caused by NH_3_-N production resulted in increased antioxidant enzyme activity in red puffer fish. Liu et al. [14] discovered that during the start of NH_3_-N stress, the juvenile golden pompano (*Trachinotus ovatus*) had an increase in antioxidant enzyme activity, and the antioxidant enzyme activity decreased when it returned to the normal environment. ROS induced by higher NH_3_-N concentrations were not eliminated by the organisms after 48 h. Chen et al. [25] found that snapping turtle (*Chelydra serpentina*) exposed to high ambient NH_3_-N had a high level of basic antioxidant defense, that most likely relied mostly on the GSH system to eliminate extra ROS. GSH/GSSG is also considered one of the essential antioxidant systems in cells, and its stable state is crucial for maintaining the normal physiological functions of cells. NH_3_-N stress transport resulted in a decline in GSH content and a gradual rise in GSSG content in the liver of seabass in the current study, which is due to GSH being converted to GSSG by GSH-Px and GST in order to scavenge intracellular free radicals and peroxides, thus maintaining normal cell and tissue function and metabolism [23]. After 48 h of NH_3_-N stress transport, GR activity increased significantly and subsequently decreased. However, the increase in GSSG content was still higher than the decrease in GSH content, indicating that the increase in GR did not offset the GSH produced by GST and GSH-Px. After a period of recovery, GSH and GSSG did not reach their initial states and the glutathione system had not recovered for short-time equilibrium.

Molecular chaperones, such as Hsp, play an essential role in preventing cellular damage from oxidative stress by ensuring the correct folding of proteins to regulate cellular homeostasis [26]. High NH_3_-N promotes the production of Hsp90 and Hsp70, according to studies [27], which suggests that the buildup of too much ROS and denatured proteins causes the elevation of Hsp levels during NH_3_-N stress. According to the study’s findings, Hsp70 and Hsp 90 levels were significantly elevated during NH_3_-N stress transport compared to CK. These findings are in line with the patterns of Hsp70 and Hsp90 gene expression after NH_3_-N exposure in kuruma shrimp (*Marsupenaeus japonicus*) and European seabass (*Dicentrarchus labrax*) [27,28]. Hsp has a key function in the prevention of protein synthesis, repair and degradation as well as apoptosis in Japanese seabass exposed to NH_3_-N stress. Hsp upregulation may be an effective measure to protect cells from oxidative stress [29].

### 4.2. The Impact of NH_3_-N Stress Transport on Liver Tissue Damage in Japanese Seabass

Excess ROS produced by NH_3_-N stress are not scavenged in time and are responsible for lipid peroxidation, inflammation and cellular damage [30]. The accumulation of ROS generates lipid peroxides (e.g., MDA) that cause cellular damage [31]. MDA levels increase after NH_3_-N stress transport and reflect the NH_3_-N generated in the liver of Japanese seabass oxidative stress, which can lead to liver damage. Peacock fish (*Poecilia reticulate*) were shown to exhibit elevated levels of ROS and MDA as well as altered antioxidant enzyme activity during NH_3_-N stress in a study by Zhang et al. [32]. However, the MDA content started to decrease after 24 h of recovery, showing that the harm caused by NH_3_-N stress transport was being gradually counteracted. After a period of recovery, the transport-induced tissue damage caused by NH_3_-N stress can revert to its original state.

The toxicity of a substance can be determined by measuring elevated AST and ALT activity, which are important markers of tissue damage [22,33]. Ali Taheri Mirghaed et al. [34] found the AST and ALT levels increased in common carp (*Cyprinus carpio*) after 24 h of NH_3_-N stress. Zhao et al. [35] found that NH_3_-N stress exposure resulted in elevated ALT and AST levels, indicating NH_3_-N stress caused tissue damage in juvenile yellow catfish (*Pelteobagrus fulvidraco*). In comparison to the CK group, the HN group’s liver was much darker and more congested. NH_3_-N poisoning can cause changes in the color and appearance of the liver during NH_3_-N stress [36,37]. NH_3_-N stress transport resulted in hepatotoxicity, which was characterized by histopathological lesions, such as vacuolation of hepatocytes, dilated hepatic sinusoids, indistinguishable cell contours, deviated nuclei, and nuclei disintegration. The damage to liver tissue was significantly reduced through photograms and histopathological optical microscopy, but it was not enough to recover to the initial level within 48 h. This indicates that the damage caused by NH_3_-N toxicity is reversible. Furthermore, Yan et al. [23] found that tissue sections of bighead carp (*Aristichthys nobilis*) after nitrite stress also revealed that liver damage caused by nitrite toxicity could be alleviated to some extent after a recovery period.

### 4.3. The Impact of NH_3_-N Stress Transport on Immunological and Inflammatory Responses in Japanese Seabass

The immunological response is a self-protective mechanism for fish against various infections. To combat infectious challenges, fish have evolved different immune systems, including lysozyme and complement. LZM and IgM maintain a healthy state in fish by eliminating invading bacteria and other pathogens while activating the complement system [38]. Studies have shown that a variety of environmental pollutants can inhibit complement C3, complement C4, IgM levels and LZM activities, thus affecting the immunological parameters of scleractinian fish [14,39]. Esam et al. [11] found the mRNA levels of complement C3 and LZM decreased, resulting in immunotoxicity when the combined ammonia- nitrogen and heat stress was applied to Nile tilapia (*Oreochromis niloticus*). In our study, complement C3, complement C4, IgM levels and LZM activities of seabass decreased after 48 h NH_3_-N stress transport, indicating a continued decline in immunological function. Gao et al. [40] also found that Takifugu rubripes (*T. rubripes*) showed a considerable decline in complement C3, complement C4, IgM levels and LZM activities after 12 h of NH_3_-N stress at different concentrations. After 24 h of recovery, the immunological parameters increased due to the weakening of oxidative stress and the gradual recovery of the immunological system from the toxic environment.

Fish that are under oxidative stress develop an inflammatory reaction. To control inflammation, TLR activation triggers the expression and release of cytokines. The results of this research concur with those of Gao et al. [41], who discovered that NH_3_-N exposure raised the gene level of *TLR-3* and activated the *TLR-3* signaling pathway. *TNF-α* is regarded as a crucial component of the TNF family, which causes inflammation and stimulates the immune system. The activated *TNF-α* could activate *NF-κB* channels. *NF-κB*, as a signaling factor, can activate several pro-inflammatory factors, including *IL-6* and *IL-1β*. In addition, The *NF-κB* pathway can also be activated by an excessive buildup of ROS, which can worsen the inflammatory response. Our experimental results suggest that both NH_3_-N stress and transport stress activated the *NF-κB* inflammatory pathway to exert anti-inflammatory effects. This was in line with previous studies on gibel carp (*Carassius gibelio*) and guppies (*Poecilia reticulate*), where it was discovered that NH_3_-N stress induced the expression of genes linked to inflammation, resulting in an inflammatory response [32,42]. Furthermore, we found that the different timing of peak gene expression of anti-inflammatory factors may vary depending on the NH_3_-N concentration.

### 4.4. The Impact of NH_3_-N Stress Transport on Apoptosis in Japanese Seabass

In addition to inducing inflammation, oxidative stress also leads to apoptosis. The oncogene *P53* is a crucial regulator of apoptosis, and ROS production brought on by NH_3_-N stress can modulate the activity of *P53*. In response to stress, *P53* alters the expression of *Bcl2* and *Bax* to induce apoptosis. The increase in the ratio of *Bax* to *Bcl2* can be used to determine the apoptosis of the organism [1]. In the current research, the *Bax/Bcl2* ratio increased in all experimental groups during NH_3_-N stress transport, suggesting that NH_3_-N stress transport could cause cell apoptosis via the *P53-Bax-Bcl2* pathway. To identify stress-induced apoptosis, Caspase expression is a crucial marker. According to Li et al. [43], that NH_3_-N exposure upregulated genes *Caspase 3* and *Caspase 9* and induced cell apoptosis. The current work showed that the expression of *Caspase 9* and *Caspase 3* was raised by both NH_3_-N exposure and transport stress, which also suggested that Caspase-dependent apoptotic pathways might be implicated in the cell death generated by transport stress of NH_3_-N.

The results of TUNEL staining showed green fluorescent spots, which verified the hypothesis that NH_3_-N stress transport leads to apoptosis. The *P53-Bax/Bcl2* apoptotic route and the Caspase-dependent apoptotic pathway may both be affected by NH_3_-N stress in Yellow catfish (*Pelteobagrus fulvidraco*) [36]. This is in line with what the current experimental study’s findings show. These findings also imply that oxidative stress and inflammation may be intimately related to cell apoptosis brought on by simultaneous exposure to these two stressors.

## 5. Conclusions

This research was the first to investigate the influence of NH_3_-N on oxidative stress, apoptosis, tissue damage and immunological response in Japanese seabass during keep-live transport. The histopathological and TUNEL results confirmed that ammonia and nitrogen stress during transport could produce ROS, resulting in oxidative stress, and that eliminated ROS through the glutathione system, but this system failed to provide adequate protection when exposed to highly oxidizing conditions. Excess ROS was involved in NH_3_-N stress-induced apoptosis through the *P53-Bax-Bcl2* pathway and the cysteine-dependent apoptosis pathway and induced an inflammatory response via the *NF-κB* pathway. These defenses declined during the recovery period, but did not return to the normal levels compared to CK. The findings of this research help towards a thorough understanding of how environmental factors effect seabass during keep-live transport, which helps towards developing optimal procedures for transport and improving the survival rate during transport.

## Figures and Tables

**Table 1:** Experimental groups and actual NH_3_-N concentration.

Experimental Group	Actual NH_3_-N Concentration (mg/L)
Control group (CK)	0.3
Low NH_3_-N group (LA, 6.4 mg/L)	6.5
Medium NH_3_-N group (MA, 12.8 mg/L)	11.5
High NH_3_-N group (HA, 19.2 mg/L)	19.0

**Table 2:** The reaction parameters for quantitative PCR.

Stage	Temperature and Time	Process
Stage 1	95 °C, 30 s	predenaturation
Stage 2	95 °C, 15 s	denature
	60 °C, 30 s (40 cycles)	annealing/extension
Stage 3	65 °C → 95 °C	melting curve: every time the temperature rises by 0.5 °C, the fluorescence signal is collected

**Table 3:** Primer sequence for quantitative PCR.

Gene	Primer Sequence (5′–3′)	Product Length (bp)	Temperature (°C)	Efficiency (%)	References
Apoptosis gene					
Actin	F: CAACTGGGATGACATGGAGAAG	200	60	101	[16]
	R: TTGGCTTTGGGGTTCAGG				
*TNF-α*	F: GACTCCATAGGCAGCAAAGC	205	60	103.2	[17]
	R: AGAAAGTCTTGCCCTCGTCA				
*IL-1β*	F: CTGAACATCAAGGGCACAGA	192	60	92.8	[17]
	R: GTTGAAGGGGACAGACCTGA				
*IL-6*	F: TACAATGTCCTCCTCAAGCACG	129	60	98.4	[18]
	R: GCCTTTGACCTCCTCCATCAG				
*TLR-3*	F: GGCCTGGATCAAATTCAAGA	139	60	103	[19]
	R: GACAGGGGACTGAATGGAGA				
*NF-κB*	F: GAAGGTATGGGAGGAGGAGTTT	189	60	102	[20]
	R: AACCACAGGGTCCAGAGGAAA				
Pro-inflammatory factor					
*P53*	F: ACCATCCTGCTGAGCTTCAT	178	60	95.3	[20]
	R: GCCCAAAACAAGTCCCTCTG				
*Caspase 3*	F: ATCACAGCAACTACGCCTCATTCG	176	60	98.9	[17]
	R: GCCTCTGCAAGCCTGGATGAAG				
*Caspase 9*	F: TGCGGAGGAGGTGAACGAGAC	138	60	90.5	[17]
	R: CGGTTCGTCGGACATGCTCAG				
*Bax*	F: GCTCCAAAGGATGATAAACGAC	182	60	96.7	[20]
	R: AACAGTGCAACCACCCGAC				
*Bcl2*	F: GTGGGGCTCTTCGCTTTTG	191	60	95.1	[21]
	R: CCATCCTCCTTGGCTCTGGA				

**Table 4:** The damage of liver records of Japanese bass transported under different NH_3_-N stress was evaluated semi-quantitatively.

Time	Groups	HPV	HSD	PN	K	CI
0 h	CK	−	−	−	−	−
48 h	CK	+	+	+	+	+
	LN	++	++	+	+	+
	MN	+++	++	+	+	+
	HN	+++	+++	++	++	++
R24 h	CK	+	+	+	−	−
	LN	+	+	+	−	−
	MN	++	+	+	+	+
	HN	++	++	+	+	+

(−) no histopathology; histopathology of (+) <20% visual field; (++) 20–60% visual field histopathology; histopathology in (+++) >60% visual field. R means recovery.

## Data Availability

The data presented in this study are available upon request from the corresponding author.
